# Limited versus extended cocaine intravenous self‐administration: Behavioral effects and electrophysiological changes in insular cortex

**DOI:** 10.1111/cns.13469

**Published:** 2020-10-29

**Authors:** Yi‐Xiao Luo, Donald Huang, Changyong Guo, Yao‐Ying Ma

**Affiliations:** ^1^ Department of Pharmacology and Toxicology Indiana University School of Medicine Indianapolis IN USA; ^2^ Department of Psychology Behavioral Neuroscience Program State University of New York Binghamton NY USA; ^3^ Stark Neurosciences Research Institute Indiana University School of Medicine Indianapolis IN USA

**Keywords:** cocaine, drug seeking, drug taking, whole‐cell patch‐clamp, insula cortex

## Abstract

**Aims:**

Limited vs extended drug exposure has been proposed as one of the key factors in determining the risk of relapse, which is the primary characteristic of addiction behaviors. The current studies were designed to explore the related behavioral effects and neuronal alterations in the insular cortex (IC), an important brain region involved in addiction.

**Methods:**

Experiments started with rats at the age of 35 days, a typical adolescent stage when initial drug exposure occurs often in humans. The drug‐seeking/taking behaviors, and membrane properties and intrinsic excitability of IC pyramidal neurons were measured on withdrawal day (WD) 1 and WD 45‐48 after limited vs extended cocaine intravenous self‐administration (IVSA).

**Results:**

We found higher cocaine‐taking behaviors at the late withdrawal period after limited vs extended cocaine IVSA. We also found minor but significant effects of limited but not extended cocaine exposure on the kinetics and amplitude of action potentials on WD 45, in IC pyramidal neurons.

**Conclusion:**

Our results indicate potential high risks of relapse in young rats with limited but not extended drug exposure, although the adaptations detected in the IC may not be sufficient to explain the neural changes of higher drug‐taking behaviors induced by limited cocaine IVSA.

## Graphical abstract

**Effects of Limited vs Extended Cocaine Exposure**: A, Timeline of cocaine‐seeking and cocaine‐taking test with specified age of days at each stage. Rats were trained by limited (upper line) or extended (lower line) cocaine intravenous self‐administration (IVSA) procedures. P, postnatal days. B, Cocaine‐taking behaviors tested by multiple dose of cocaine (ie, 0.1, 0.3, 1.0, 3.0 mg/kg per infusion) on withdrawal days (WD) 46‐48 after IVSA. C, cocaine (0.1 mg/kg per infusion)‐taking behaviors of individual rats are represented by the circles in the columns. D, Sample trace of action potential from the pyramidal neurons in the IC 1 day (left) and 45 days (right) after limited saline (denoted “S”)/ cocaine (denoted “C”) IVSA training. E, Summarized data showing that, relative to the saline controls, limited cocaine treatment significantly prolonged the rise time of action potential 45 days but not 1 day after IVSA training.

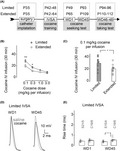

## 1 INTRODUCTION

Addiction is a mental disorder defined by compulsive and chronic drug taking. The development of drug abuse after an extended period of drug taking has been suggested to depend on a shift from early recreational drug use to later compulsive drug abuse. The neurobiological mechanisms leading to this shift remain unknown. Furthermore, compared with a limited drug history, extended drug exposure was assumed to have a higher probability to lead to prolonged neuronal alterations, resulting in a higher risk of drug relapse and a further extension of drug abuse.^1^, ^2^, ^3^, ^4^

The insular cortex (IC), or insula for short, plays an important role in drug addiction. Both human^5^, ^6^, ^7^ and animal studies^8^ showed that IC lesions interrupt addictive behaviors, suggesting that the IC or the IC‐mediated interoceptive system is sensitized in addicted subjects as a consequence of addiction history and a cause of relapse. However, a different picture was suggested by neuroimaging studies, in which reduced activity^9^, ^10^ and shrunken gray matter^5^, ^11^ in the IC were detected in individuals with substance abuse history. These apparently contradictory lines of evidence may indicate that the IC can both promote drug use (eg, via increased perception of craving for drugs) and weaken the processes that prevent drug use (such as decision‐making and the evaluation of negative consequences).^12^ The IC consists of 3 subregions, that is, posterior granular insular, anterior agranular insula (AAI), and the intermediate dysgranular insula).^13^, ^14^, ^15^ Particular attention in addiction research has been payed to the AAI due to its rich chemoarchitecture, including the presence of dopamine and opioid receptors.^6^, ^16^, ^17^, ^18^, ^19^, ^20^

The present study used a rat model of cocaine intravenous self‐administration (IVSA) to explore the behavioral and electrophysiological consequences of limited vs extended IVSA after short (1 day) or prolonged (45 days) withdrawal periods (designated as WD1 and WD45, respectively). Experiments started with rats at the age of 35 days, a typical adolescent stage when initial drug exposure occurs often in humans.^21^, ^22^

## 2 MATERIALS AND METHODS

### 2.1 Experimental subjects

**Figure 1:**
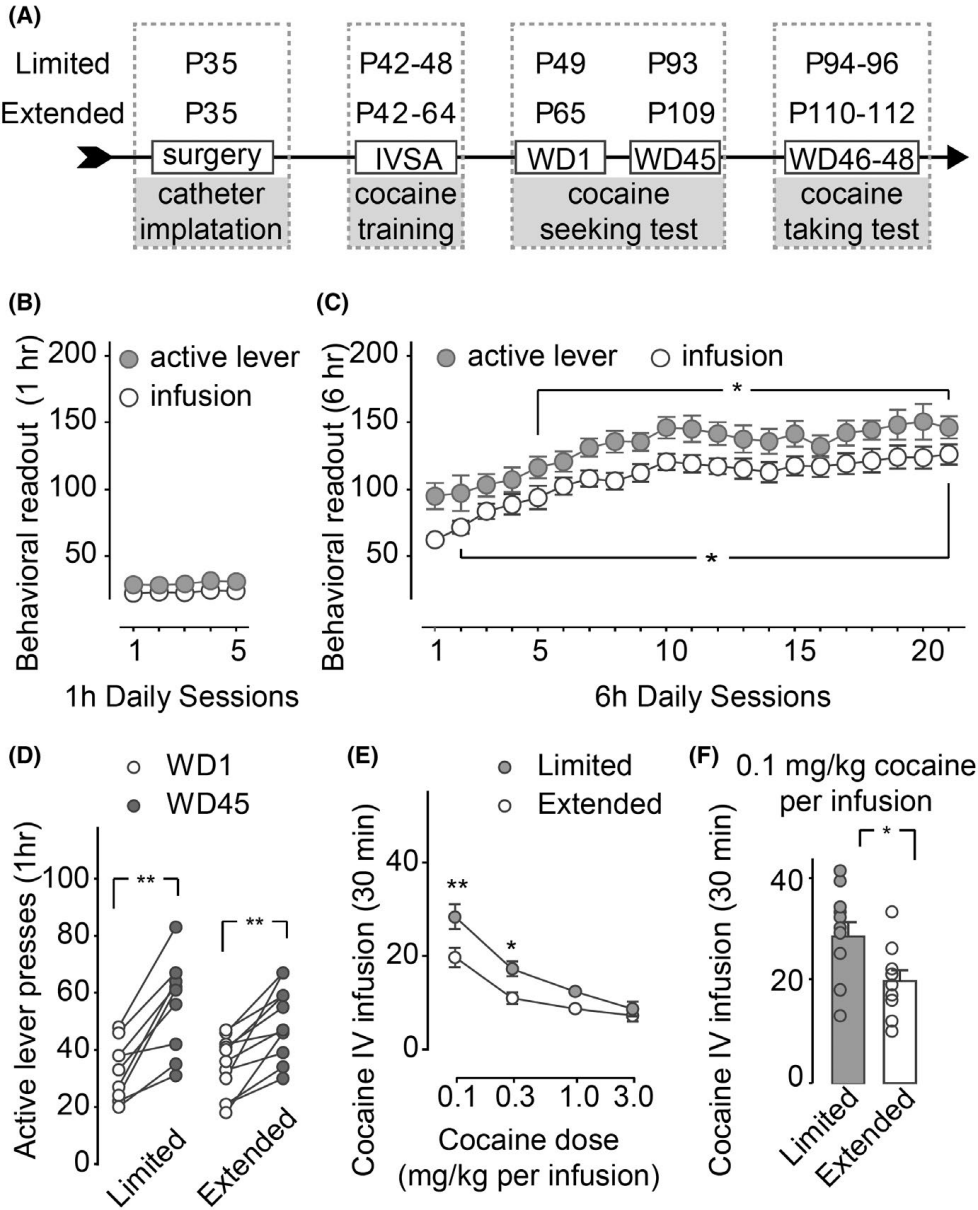
Effects limited vs extended cocaine IVSA on cocaine‐seeking and cocaine‐taking behaviors during the withdrawal period. A, Timeline of cocaine‐seeking and cocaine‐taking test with specified age of days at each stage. Rats were trained by limited (upper line) or extended (lower line) cocaine IVSA procedures. P, postnatal days. B, The daily # of cocaine IV infusion and daily # of active lever press by rats exposed to limited cocaine IVSA procedure. C, The daily # of cocaine IV infusion and daily # of active lever press by rats exposed to extended cocaine IVSA procedure. D, Cocaine‐taking behaviors on WD1 and WD45. E, Cocaine‐taking behaviors tested by multiple dose of cocaine (ie, 0.1, 0.3, 1.0, 3.0 mg/kg per infusion) on WD46‐48. F, cocaine (0.1 mg/kg per infusion)‐taking behaviors of individual rat are represented by the circles in the columns. Data were analyzed by one‐way ANOVA (each curve in B, C) and two‐way ANOVA with repeated measures (D,E), followed by Bonferroni post‐hoc tests or Student’s *t* test (F). n = 11 in limited cocaine IVSA group; n = 10 in extended cocaine IVSA group. *P < .05; **P < .01, comparations between day 1 vs any of other days during the IVSA training (C), WD1 vs WD45 (D), or limited vs extended IVSA groups (E,F)

All procedures were performed in accordance with the United States Public Health Service Guide for Care and Use of Laboratory Animals and were approved by the institutional Animal Care and Use committee at the State University of New York, Binghamton and Indiana University School of Medicine. Experiments were conducted on male Sprague Dawley rats, bred in‐house using breeders originally derived from Envigo (USA). With the day of birth being deemed as postnatal day (P) 0, rats were allowed to develop normally until P21‐23 when animals were weaned and pair‐housed in standard Plexiglas bins unless receiving cannula implantation procedure. Rats were maintained on a 7 am/7 pm light/dark schedule with *ad libitum* access to food and water. Rats were allowed 5‐7 days to acclimate to colony conditions and handled to habituate them to human contact prior to experimentation. Similar to our previous studies, rats received catheter implantation on postnatal ~35 days. Seven days later, rats received one overnight session, followed by 5 of 1‐hour daily sessions (considered as limited training procedure) or 21 of 6‐hour daily sessions (considered as extended training procedure) of cocaine IVSA training.^23^, ^24^ In total, 75 rats were used in this study, among which 8 rats were excluded due to patency failure of the catheter (n = 6) and behavioral outliers (n = 2). From the 67 remaining subjects, 21 were trained by limited (n = 11) or extended (n = 10) cocaine IVSA procedures, followed by cocaine‐seeking test on WD1 and WD45, and cocaine‐taking test on WD46‐48 (Figure 1A). The other 46 rats were trained by limited (n = 9 for saline and n = 14 for cocaine) or extended (n = 10 for saline and n = 13 for cocaine) IVSA procedures and whole‐cell patch‐clamp recordings were obtained either 1 or 45 days after the last IVSA session.

### 2.2 Intravenous self‐administration of cocaine

#### 2.2.1 Catheter implantation

As described previously,^23^, ^24^, ^25^, ^26^, ^27^ a silastic catheter was inserted into the right jugular vein, and the distal end was led subcutaneously to the back between the scapulae. Catheters were constructed from silastic tubing (~8 cm; inner diameter 0.020 in, outer diameter 0.037 in) connected to a One Channel Rat Button (Instech Labs). Rats were allowed to recover for ~7 days. During recovery, the catheter was flushed daily with 1 mL/kg body weight of heparin (10 U/mL) and gentamicin antibiotic (5 mg/mL) in sterile saline to help protect against infection and catheter occlusion.

#### 2.2.2 Self‐administration apparatus

Experiments were conducted in operant‐conditioning chambers enclosed within sound‐attenuating cabinets (Med Associates). Each chamber contained two levers randomly assigned as active vs inactive levers, a food dispenser, and the conditioned stimulus (CS) light 9 cm above each lever. No food or water was provided in the chambers during the training or testing sessions.

#### 2.2.3 Intravenous cocaine self‐administration training

*~7 days after catheter implantation, c*ocaine self‐administration training began with an overnight session at 7 pm to 7 am on the following day. The daily training session, either 1 hour per day for 5 days as limited cocaine access or 6 hours per day for 21 days as extended cocaine access, started on the day after. The same training protocol was used in overnight and daily sessions. Rats were placed in the self‐administration chamber on a fixed ratio (FR) 1 reinforcement schedule with the house light on. Active lever press resulted in a cocaine infusion (0.75 mg/kg over 2‐4 seconds) and illumination of a CS light above the active lever for 20 seconds with the house light off. In contrast, the inactive lever press led to no outcome but was also recorded. Rats that received at least 60 cocaine rewards in the overnight session were allowed to move to daily self‐administration of cocaine ~24 hr after the overnight training on an FR1 reinforcement schedule. Animals that did not meet this standard (n = 1) were removed from subsequent self‐administration training.

#### 2.2.4 Cocaine‐seeking test

Rats previously assigned for behavioral test were tested on both WD1 and WD45 for their cocaine‐seeking behaviors. The cocaine‐seeking test lasted for 1 hour, during which rats were re‐exposed to the operant chamber with the exact same conditions as during cocaine IVSA training except no cocaine IV delivery was programmed after active lever press. The active lever press during 1 hour seeking test was analyzed as an index of cocaine‐seeking level (Figure 1D).

#### 2.2.5 Cocaine‐taking test

Multiple doses of cocaine were evaluated in the cocaine‐taking test as described before.^28^ Briefly, a 2‐hour period was divided into four 30‐minute segments, which allowed the assessment of four pseudo‐randomized doses of cocaine (0.1, 0.3, 1.0, and 3.0 mg/kg per injection) in a 2‐hour session. Two adjacent 30‐minute sessions were spaced by a ~5‐minute interval. During each 30‐minute session, the exact same conditions, although the dose of cocaine per injection was different, were applied to rats, which means rats received cocaine IV delivery after active lever press, followed by a 20‐second timeout during which the cue light signals were presented and no cocaine delivery was allowed. This 2‐hour daily session was done once per day for 3 consecutive days (ie, WD46‐48). The average number of cocaine infusions from 3 30‐minute sessions with a specific dose of cocaine was analyzed as an index of cocaine‐taking level.

### 2.3 Brain slice whole‐cell patch‐clamp recordings

Standard procedures were used for preparing slices and whole‐cell patch‐clamp recordings as detailed in our previous publications.^23^, ^24^, ^27^ Before sacrifice, the rats were anesthetized with isoflurane and subsequently transcardially perfused with 4°C cutting solution (in mM: 135 N‐methyl‐D‐glucamine, 1 KCl, 1.2 KH_2_PO_4_, 0.5 CaCl_2_, 1.5 MgCl_2_, 20 choline‐HCO_3_, 11 glucose, pH adjusted to 7.4 with HCl, and saturated with 95% O_2_/5% CO_2_). The rat was decapitated, and then the brain was removed and glued to a block before slicing using a Leica VT1200s vibratome in 4°C cutting solution. Coronal slices of 250‐µm thickness were cut such that the preparation contained the signature anatomical landmarks (eg, the rhinal fissure, the anterior commissure, and the corpus callosum) that clearly delineate the AAI area. After allowing at least 1 hour for recovery, slices were transferred from a holding chamber to a submerged recording chamber where it was continuously perfused with oxygenated ACSF maintained at 30 ± 1°C.

Standard whole‐cell current‐ or voltage‐clamp recordings were obtained with a MultiClamp 700B amplifier (Molecular Devices), filtered at 3 kHz, amplified 5 times, and then digitized at 20 kHz with a Digidata 1550B analog‐to‐digital converter (Molecular Devices). The recording electrodes (3‐5 MΩ) were filled with (in mmol/L): 108 KMeSO_3_, 20 KCl, 0.4 K‐EGTA, 10 HEPES, 2.5 Mg‐ATP, 0.25 Na‐GTP, 7.5 phosphocreatine (Na2), 1 L‐glutathione, 2 MgCl_2_, pH 7.3. The recording bath solution contained (in mM): 119 NaCl, 2.5 KCl, 2.5 CaCl_2_, 1.3 MgCl_2_, 1 NaH_2_PO_4_, 26.2 NaHCO_3_, and 11 glucose, saturated with 95% O_2_/ 5% CO_2_ at 30 ± 1°C. Details for whole‐cell patch‐clamp recordings can be found in one of our previous publications.^27^ Cells were patched in voltage clamp mode and held at −70 mV. Cell membrane capacitance (Cm), input resistance (Rm), and time constant (τ) were calculated by applying a depolarizing step voltage command (5 mV) and using the membrane test function integrated in the pClamp10 software. Then, recordings were switched to current clamp mode. Resting membrane potential was measured and then adjusted to −70 mV through injection of positive current (50‐100 pA). The intrinsic excitability was examined using a series of depolarizing current pulses and by constructing input‐output (I‐O) functions.

### 2.4 Data acquisition and analysis

**Figure 2:**
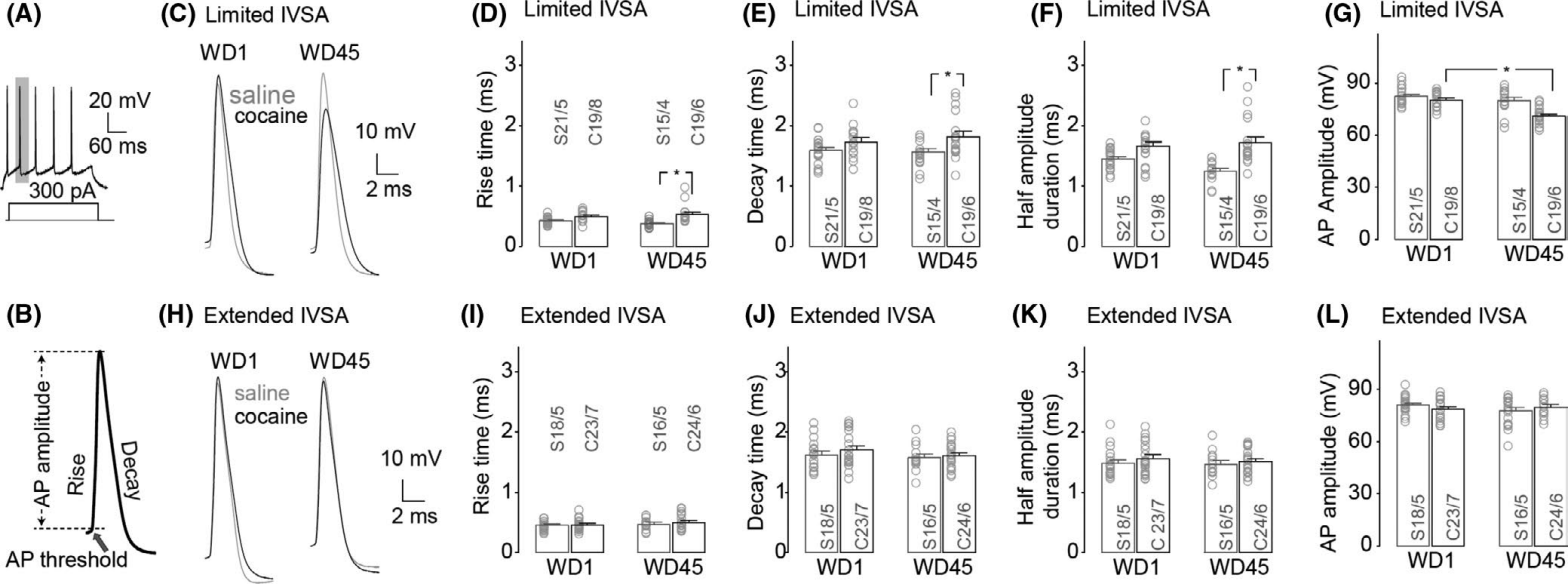
Effects of limited vs extended cocaine IVSA on the kinetics of action potentials. A, A sample trace showing the second action potential induced by 300 pA injection current was analyzed for the spike kinetics. B, A sample trace of action potential showing the measurement of rise time, decay time, and amplitude. C, Sample trace of action potential from the pyramidal neurons in the IC 1 d (left) and 45 d (right) after limited saline (denoted “S”)/ cocaine (denoted “C”) IVSA training. D, E, Summarized data showing that, relative to the saline controls, limited cocaine treatment significantly prolonged the rise time (D), decay time (E), and half‐amplitude duration (F) of action potential 45 d but not 1 d after IVSA training. G, Summarized data showing that limited cocaine treatment significantly decreased the amplitude of action potential on WD45, relative to WD1. H, Sample trace of action potential from the pyramidal neurons in the IC 1 d (left) and 45 d (right) after extended saline (in gray)/ cocaine (in black) IVSA training. I‐L, Summarized data showing no difference between saline controls vs extended cocaine‐treated rats in rise time (I), decay time (J), half‐amplitude duration (K), or amplitude (L) of action potential on both 1 d and 45 d after IVSA training. Data were analyzed by two‐way ANOVA, followed by Bonferroni post‐hoc tests. n/m, the number of cells/animals for data collection. *P < .05

**Figure 3:**
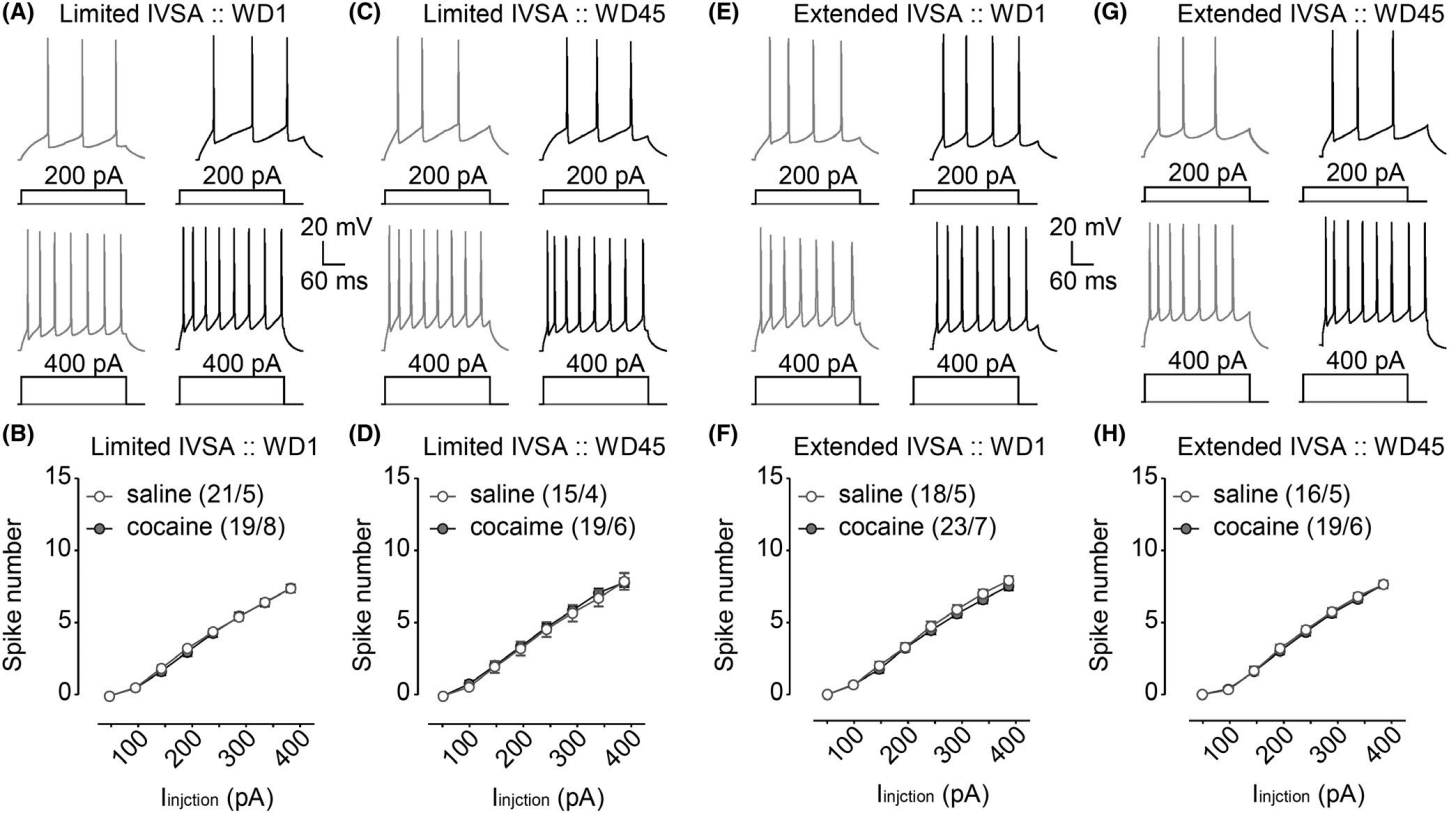
Effects of limited vs extended cocaine IVSA on spike number of pyramidal neurons in the IC induced by current injections. A, B, Example traces (A) and summarized data (B) showing no significant effects of limited cocaine IVSA on spike number 1 d after IVSA training. C, D, Example traces (C) and summarized data (D) showing no significant effects of limited cocaine IVSA on spike number 45 d after IVSA training. E, F, Example traces (E) and summarized data (F) showing no significant effects of extended cocaine IVSA on spike number 1 d after IVSA training. G, H, Example traces (G) and summarized data (H) showing no significant effects of extended cocaine IVSA on spike number 45 d after IVSA training. Data were analyzed by two‐way ANOVA with repeated measures, followed by Bonferroni post‐hoc tests. n/m, the number of cells/animals for data collection

**Figure 4:**
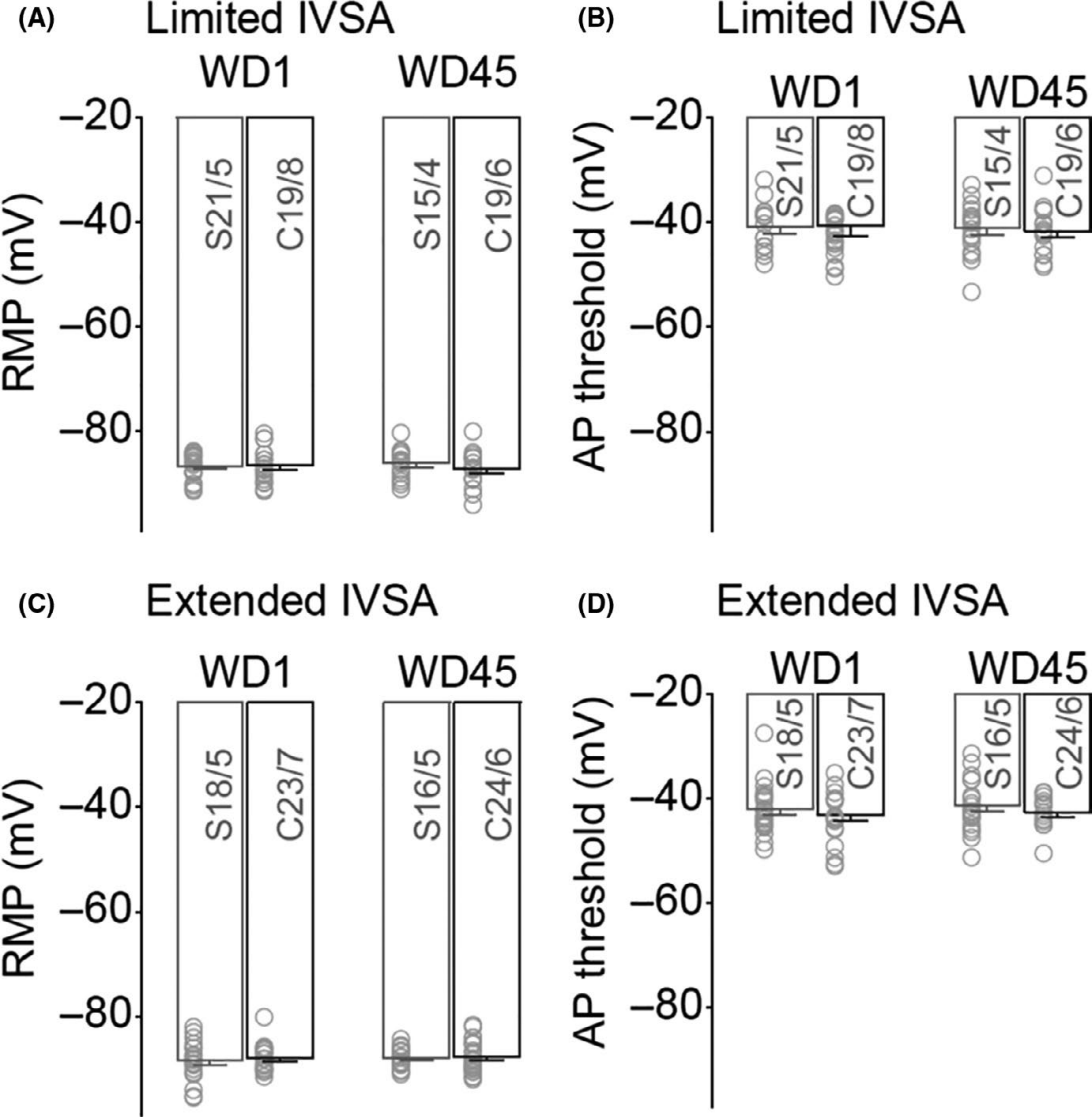
Effects of limited vs extended cocaine IVSA on RMP and action potential threshold. A, Summarized data showing no significant effects of limited IVSA on RMP in insula pyramidal neurons. B, Summarized data showing no significant effects of limited IVSA on action potential threshold in insula pyramidal neurons. C, Summarized data showing no significant effects of extended IVSA on RMP in insula pyramidal neurons. D, Summarized data showing no significant effects of limited IVSA on action potential threshold in insula pyramidal neurons. Data were analyzed by two‐way ANOVA, followed by Bonferroni post‐hoc tests. n/m, the number of cells/animals for data collection

Data were collected either 2 days or 21 days after the last IVSA session. All results are shown as mean ± SEM. Each experiment was replicated in at least 10 rats for *in vivo* behavioral tests and at least 4 rats (usually 2‐4 cells per animal were recorded per group) for *in vitro* electrophysiological recordings. Sample size is presented as n, referring to the number of rats in behavioral tests, or presented as m/n, where “m” refers to the number of cells examined and “n” refers to the number of rats used for electrophysiology experiments. Statistical significance was assessed using the Student *t* test (Figure 1F), one‐way ANOVA with repeated measures (each individual curve in Figure 1B,C), two‐way ANOVA (Figures 2D‐G,I‐L and 4A‐D), or two‐way ANOVA with repeated measures (Figures 1D,E and 3B,D,F,H), followed by Bonferroni post‐hoc tests. The analytical unit is the animal in behavioral data (Figure 1), or both the cell and the animal in the *in vitro* slice recording data (Figure 2, 3, 4).

## 3 RESULTS

### 3.1 Effects of limited vs extended cocaine IVSA on cocaine‐seeking and cocaine‐taking behaviors during the withdrawal period

Adolescent rats (n = 21), implanted with catheter on postnatal day 35 and recovered for ~1 week, were trained by limited (ie, 1 hour per day for 5 days) or extended (ie, 6 hours per day for 21 days) cocaine IVSA procedure, followed by a period of forced abstinence during which the rats were just housed in the home cage, except on WD1 and WD45 when they underwent the cocaine‐seeking test, and on WD46‐48 when they received the cocaine‐taking test in the operant chambers as shown in Figure 1A. A flat curve of the daily cocaine infusion (*F*_4,40_ = 1.4, *P* = .24) and the number of active lever presses (*F*_4,40_ = 2.2, *P* = .09) was observed during the 5 day of limited cocaine IVSA training sessions (Figure 1B). An increasing number of daily cocaine infusions (*F*_20,180_ = 24.9, *P* < .01) and active lever presses (*F*_20,180_ = 8.0, *P* < .01) were observed during 21‐day extended cocaine IVSA sessions (Figure 1C). During the withdrawal period, the cocaine‐seeking behaviors were increased on WD45, compared with that on WD1, regardless of the limited vs extended cocaine history (limited/extended *F*_1,18_ = 0.2, *P* = .20; WD1/WD45 *F*_1,18_ = 62.4, *P* < .01; limited/extended × WD1/WD45 interaction *F*_1,18_ = 2.0, *P* = .17; Figure 1D). Interestingly, rats with a history of limited cocaine IVSA showed an upward shift of dose‐response curve (ie, the dose of cocaine and the cocaine‐taking response), compared with that from the extended group (limited/extended × cocaine dose interaction *F*_3,57_ = 3.0, *P* = .04; limited/extended *F*_1,19_ = 7.9, *P* = .01; cocaine dose *F*_3,57_ = 60.4, *P* < .01; Figure 1E), partially demonstrated at the dose of 0.1 mg/kg cocaine per infusion (*t*_19_ = 2.5, *P* = .02; Figure 1F). Drug‐seeking and drug‐taking behaviors after a prolonged withdrawal period have been well accepted as reliable indices of relapse.^29^, ^30^, ^31^ Thus, we conclude that the extended cocaine IVSA procedure used in the current study did not increase the risk for relapse. Interestingly, the limited cocaine IVSA procedure may increase the relapse risk due to significantly increased drug‐taking behaviors on WD45.

### 3.2 Effects of limited vs extended cocaine IVSA on the kinetics and amplitude of evoked action potentials in insula pyramidal neurons

Whole‐cell current clamp recordings in IC‐containing coronal slices were performed in rats 1 day or 45 days after IVSA of saline or cocaine. The kinetics and amplitude of the action potential were analyzed by measuring the 2nd action potential induced by 300 pA current injection (Figure 2A). The insula pyramidal neurons from rats with a history of limited IVSA procedure, that is, 1 hour per day for 5 days, displayed significantly increased rise time (Figure 2D, saline/cocaine × WD1/WD45 interaction *F*_1,65_ = 4.1, *P* = .04, saline/cocaine *F*_1,65_ = 21.3, *P* < .01, and WD1/WD45 *F*_1,65_ = 0.1, *P* = .80, cell‐based; saline/cocaine × WD1/WD45 interaction *F*_1,19_ = 4.4, *P* = .049, saline/cocaine *F*_1,19_ = 12.1, *P* < .01, and WD1/WD45 *F*_1,19_ = 0.1, *P* = .66, animal‐based), decay time (Figure 2E, saline/cocaine × WD1/WD45 interaction *F*_1,65_ = 4.2, *P* = .04, saline/cocaine *F*_1,65_ = 30.0, *P* < .01, and WD1/WD45 *F*_1,65_ = 4.9, *P* = .03, cell‐based; saline/cocaine × WD1/WD45 interaction *F*_1,19_ = 4.5, *P* = .047, saline/cocaine *F*_1,19_ = 27.3, *P* < .01, and WD1/WD45 *F*_1,19_ = 4.5, *P* = .047, animal‐based), half‐amplitude duration (Figure 2F, saline/cocaine × WD1/WD45 interaction *F*_1,65_ = 4.7, *P* = .03, saline/cocaine *F*_1,65_ = 36.4, *P* < .01, and WD1/WD45 *F*_1,65_ = 3.0, *P* = .08, cell‐based; saline/cocaine × WD1/WD45 interaction *F*_1,19_ = 4.6, *P* = .045, saline/cocaine *F*_1,19_ = 24.9, *P* < .01, and WD1/WD45 *F*_1,19_ = 2.7, *P* = .12, animal‐based), and decreased amplitude of the evoked action potential (Figure 2G, saline/cocaine × WD1/WD45 interaction *F*_1,65_ = 14.1, *P* < .01, saline/cocaine *F*_1,65_ = 6.9, *P* = .01, and WD1/WD45 *F*_1,65_ = 9.0, *P* < .01, cell‐based; saline/cocaine × WD1/WD45 interaction *F*_1,19_ = 10.8, *P* = .01, saline/cocaine *F*_1,19_ = 6.4, *P* = .02, and WD1/WD45 *F*_1,19_ = 8.4, *P* = 0.01, animal‐based) 45 days, but not 1 day, after cocaine IVSA, compared with saline controls. However, no significant differences in rise time (Figure 2I, saline/cocaine × WD1/WD45 interaction *F*_1,68_ = 0.23, *P* = .63, saline/cocaine *F*_1,68_ = 0.5, *P* = .50, and WD1/WD45 *F*_1,68_ = 1.6, *P* = .22, cell‐based; saline/cocaine × WD1/WD45 interaction *F*_1,19_ = 0.4, *P* = .53, saline/cocaine *F*_1,19_ = 1.3, *P* = .27, and WD1/WD45 *F*_1,19_ = 0.5, *P* = .49, animal‐based), decay time (Figure 2J, saline/cocaine × WD1/WD45 interaction F_1,68_ = 0.2, *P* = .67, saline/cocaine *F*_1,68_ = 1.0, *P* = .32, and WD1/WD45 *F*_1,68_ = 1.4, *P* = .24, cell‐based; saline/cocaine × WD1/WD45 interaction *F*_1,19_ = 0.4, *P* = .54, saline/cocaine *F*_1,19_ = 0.8, *P* = .37, and WD1/WD45 F_1,19_ = 1.5, *P* = .25, animal‐based), half‐amplitude duration (Figure 2K, saline/cocaine × WD1/WD45 interaction *F*_1,68_ = 0.1, *P* = .75, saline/cocaine *F*_1,68_ = 1.2, *P* = .28, and WD1/WD45 *F*_1,68_ = 0.4, *P* = .51, cell‐based; saline/cocaine × WD1/WD45 interaction *F*_1,19_ = 0.3, *P* = .57, saline/cocaine *F*_1,19_ = 1.1, *P* = .30, and WD1/WD45 *F*_1,19_ = 0.6, *P* = .44, animal‐based), and action potential amplitude (Figure 2L, saline/cocaine × WD1/WD45 interaction *F*_1,68_ = 2.1, *P* = .15, saline/cocaine *F*_1,68_ < 0.1, *P* = .88, and WD1/WD45 *F*_1,68_ = 0.5, *P* = .49, cell‐based; saline/cocaine × WD1/WD45 interaction *F*_1,19_ = 2.3, *P* = .15, saline/cocaine *F*_1,19_ = 0.1, *P* = .42, and WD1/WD45 *F*_1,19_ = 0.4, *P* = .55, animal‐based) were detected in rats with an extended IVSA (ie, 6 hr per day for 21 days) of saline vs cocaine either 1 day or 45 days after the last IVSA training session, demonstrating significant kinetic changes of APs after a prolonged withdrawal period from limited but not extended IVSA of cocaine.

### 3.3 Effects of limited vs extended cocaine IVSA on intrinsic excitability of insula pyramidal neurons

The number of spikes evoked by depolarizing current injections ranging from 50 to 400 pA were also analyzed. Our data showed that, relative to saline controls, cocaine IVSA procedure did not affect the number of action potentials evoked 1 day vs 45 days after the last IVSA session (Figure 3). Specifically, neither significant effects of limited cocaine IVSA on spike number were detected 1 day after IVSA training (Figure 3B, saline/cocaine × I_inj_ interaction *F*_7,266_ = 0.5, *P* = .85, and saline/cocaine *F*_1,38_ < 0.1, *P* = .82, cell‐based; saline/cocaine × I_inj_ interaction *F*_7,77_ = 0.5, *P* = .83, and saline/cocaine *F*_1,11_ < 0.1, *P* = .89, animal‐based) or 45 days after IVSA training (Figure 3D, saline/cocaine × I_inj_ interaction *F*_7,224_ = 0.2, *P* = .98, and saline/cocaine *F*_1,32_ = 0.1, *P* = .77, cell‐based; saline/cocaine × I_inj_ interaction *F*_7,56_ = 0.3, *P* = .95, and saline/cocaine *F*_1,8_ = 0.1, *P* = .77, animal‐based), nor significant effects of extended cocaine IVSA on spike number were detected 1 day after IVSA training (Figure 3F, saline/cocaine × I_inj_ interaction *F*_7,273_ = 0.8, *P* = .60, and saline/cocaine *F*_1,39_ = 0.4, *P* = .55, cell‐based; saline/cocaine × I_inj_ interaction *F*_7,70_ = 1.0, *P* = .46, and saline/cocaine *F*_1,10_ = 0.4, *P* = .54, animal‐based) or 45 days after IVSA training (Figure 3H, saline/cocaine × I_inj_ interaction *F*_7,266_ = 0.2, *P* = .98, and saline/cocaine *F*_1,38_ = 0.1, *P* = .79, cell‐based; saline/cocaine × I_inj_ interaction *F*_7,63_ = 0.2, *P* = .97, and saline/cocaine *F*_1,9_ = 0.1, *P* = .80, animal‐based).

Further data analyses showed no significant difference of RMPs in rats with a history with limited (Figure 4A, saline/cocaine × WD1/WD45 interaction *F*_1,70_ = 2.2, *P* = .14, saline/cocaine *F*_1,70_ = 1.1, *P* = .30, and WD1/WD45 *F*_1,70_ = 0.1, *P* = .79, cell‐based; saline/cocaine × I_inj_ interaction *F*_1,19_ = 2.4, *P* = .14, saline/cocaine × *F*_1,19_ = 0.9, *P* = .35, and WD1/WD45 *F*_1,19_ = 0.1, *P* = .80, animal‐based) or extended (Figure 4C, saline/cocaine × WD1/WD45 interaction *F*_1,77_ = 3.3, *P* = .08, saline/cocaine *F*_1,77_ = 3.1, *P* = .08, and WD1/WD45 *F*_1,77_ = 1.0, *P* = .34, cell‐based; saline/cocaine × I_inj_ interaction *F*_1,19_ = 3.0, *P* = .10, saline/cocaine × *F*_1,19_ = 3.1, *P* = .09, and WD1/WD45 *F*_1,19_ = 1.0, *P* = .38, animal‐based) cocaine vs saline IVSA. Finally, limited (Figure 4B, saline/cocaine × WD1/WD45 interaction *F*_1,70_ = 0.1, *P* = .79, saline/cocaine *F*_1,70_ < 0.1, *P* = .97, and WD1/WD45 *F*_1,70_ = 0.2, *P* = .67, cell‐based; saline/cocaine × I_inj_ interaction *F*_1,19_ = 0.1, *P* = .78, saline/cocaine × *F*_1,19_ < 0.1, *P* = .95, and WD1/WD45 *F*_1,19_ = 0.1, *P* = .71, animal‐based) or extended (Figure 4D, saline/cocaine × WD1/WD45 interaction *F*_1,77_ = 0.3, *P* = .58, saline/cocaine *F*_1,77_ = 3.0, *P* = .09, and WD1/WD45 *F*_1,77_ = 1.6, *P* = .21, cell‐based; saline/cocaine × I_inj_ interaction *F*_1,19_ = 0.4, *P* = .54, saline/cocaine × *F*_1,19_ = 2.7, *P* = .11, and WD1/WD45 *F*_1,19_ = 1.6, *P* = .22, animal‐based) cocaine IVSA did not significantly influence the threshold of action potential. Thus, the membrane excitability of AAI pyramidal neurons was not affected by either limited or extended cocaine IVSA history.

## 4 DISCUSSION

Our data demonstrated no differences of drug‐seeking behaviors after limited vs extended cocaine IVSA, although both limited and extended IVSA rats showed higher drug‐seeking behaviors on WD45, compared with WD1, which indicates the duration of withdrawal period can significantly increase the drug‐seeking behaviors as evidenced before.^32^, ^33^, ^34^, ^35^ Importantly, limited cocaine IVSA significantly increased cocaine‐taking behaviors compared with the extended cocaine IVSA group as supported by the upward shift of the dose‐response curve of cocaine taking at the later withdrawal stage. Thus, limited, compared with extended, cocaine history, may lead to a potential high risk of relapse to cocaine. Notably, this behavioral outcome dispels the current belief that extended drug exposure has more robust effects on drug‐seeking and drug‐taking behaviors. For example, extended access to drugs significantly increased drug‐taking behaviors in the withdrawal period^36^, ^37^, ^38^ and compulsive drug‐seeking behaviors could only be established in rats by extended but not limited cocaine IVSA procedure^39^. It is critical to point out the developmental stages when drug exposure and drug withdrawal occur. Relative to adults, adolescent rats took cocaine more readily, were more sensitive to lower doses, and showed greater escalation of cocaine intake.^40^ Thus, our IVSA training was done at the adolescence stage. On another hand, it has been fashionable in addiction research to run drug exposure with extended daily sessions (≥6 hours) during a long period (eg, at least 10 days).^41^, ^42^, ^43^, ^44^ However, studies on limited vs extended drug exposure in adolescent animals were still lacking. Most, if not all, of the previous studies comparing the prolonged effects of limited vs extended drug exposure on drug‐seeking/taking behaviors used adult animals, whereas rats were adolescent when the current experiments started. We found high risk of relapse in rats with limited but not extended drugs at the adolescent stage. Thus, it is assumed the developmental stage of drug experience matters more than the amount of drug in the young brain.

This is based on several observations: **First**, at the initial drug‐taking stage, young brains may be more sensitive to the effects of drugs. This vulnerability of adolescents may be fully masked by chronic drug administration.^21^, ^22^ According to clinical observations, the onset of drug use during adolescence appears to lead to a prolonged sensitivity to drug relapse and dependence in humans.^45^, ^46^ **Second**, withdrawal experience of the young brain may trigger more intensive neuronal alterations that facilitate the drug‐seeking and drug‐taking behaviors even after a prolonged withdrawal period. Our previous works demonstrated active neuronal adaptations by the passage of the withdrawal period.^23^, ^24^, ^47^ The current limited cocaine IVSA procedure involves at least two factors, which significantly increase the risk of drug‐taking behaviors at the late withdrawal time. One is the limited exposure of cocaine, which is enough for young brains to be reshaped with the abusive potential of drugs. The other is the withdrawal period starting from the adolescent stage when the brain is highly malleable. Our current conclusion, that is, high risk of relapse associated with limited drug exposure at the adolescent stage, followed by a withdrawal period, is not contradictory but an essential complement to the established addiction theory.

Low drug taking observed in rats with a history of extended cocaine IVSA might be attributed to their poor learning and memory, which resulted in a poor retrieval of the previously learned association between the action (ie, lever press) and output (cocaine IV delivery and cue light) in the seeking/taking test during the withdrawal period. This can be excluded by the comparable drug‐seeking behaviors between rats pre‐treated by limited vs extended cocaine IVSA on either WD1 or WD45, and comparable drug‐taking behaviors at 10 mg/kg cocaine per injection on WD46‐48, indicating the retrieval of previous acquired operant behaviors was intact. Also, the poor locomotion could be a consequence of extended cocaine IVSA, which can be excluded by the comparable number of inactive lever presses in rats treated by limited vs extended cocaine IVSA (eg, active lever presses in 1‐hour drug‐seeking test on WD45: limited, 10.1 ± 3.4; extended, 8.7 ± 2.7, *P* = .34).

Our data showed escalated drug intake in rats with extended IVSA procedure, consistent with previous observations.^48^ However, there are some exceptions; data from rhesus monkeys showed no escalated intake over time after extended access to cocaine IVSA.^49^ We found that rats with escalated drug‐taking behaviors during the extended cocaine IVSA procedure did not show high risk of relapse. Together with the previous report that the presence of escalation did not affect reinstatement,^48^ we assume that the presence of escalation has limited predictability on the risks of relapse. Escalation of cocaine taking was produced by continuous cocaine IVSA.^50^

Although the critical involvement of the IC in addiction‐related behaviors has been widely accepted, little was known about the intrinsic excitability of IC pyramidal neurons in rats which experienced limited vs extended IVSA. We found some minor but significant effects of limited but not extended cocaine exposure on the kinetics and amplitude of action potentials on WD 45 after limited cocaine IVSA, in pyramidal neurons from the AAI. Although our original hypothesis was that, compared with the saline controls, extended, but not limited, cocaine exposure may lead to significant changes (eg, modified intrinsic excitability) in pyramidal neurons in the AAI, we did not see any effects of extended cocaine IVSA on this electrophysiological readout in insular pyramidal neurons. These findings indicate that limited cocaine exposure has some, probably priming, effects on the capacity of IC neurons to generate APs and these effects are compensated after extended cocaine exposure. It is worth to note that the slower kinetics and smaller amplitude of action potentials observed on WD45 after limited cocaine IVSA could be also observed in unhealthy cells. Our cells in different groups were similar in terms of access resistance and holding current evaluated by Membrane Test in voltage clamp, indicating comparable quality of cells among groups. Thus, the observed changes in kinetics and amplitude of action potentials might be attributable to the adaptations of ion channels (eg, Na^+^ and K^+^).

The involvement of the IC in cocaine‐related effects is well supported by previous literature. First, molecular alterations in the IC were detected 21 days but not 1 day after 5‐day cocaine exposure. Specifically, the cocaine challenge produced a decrease in zif 268 expression after the 21‐day, but not 1‐day, withdrawal period.^51^ Second, by using an extended procedure of cocaine access, the anterior IC was found to mediate the maintenance of escalated cocaine‐taking behaviors but not the initial development of cocaine‐taking behaviors.^52^ Third, different subregions of IC may be involved in cocaine‐seeking behaviors triggered by different factors. Agranular IC was involved in drug context‐induced reinstatement of cocaine‐seeking behavior without altering locomotor activity.^53^ Anterior dorsal agranular IC participated in cue‐associated cocaine‐seeking behaviors,^54^ whereas rostral agranular IC was reported to be related to cocaine‐seeking behaviors intensified by negative affect.^55^ Last but not least, human studies also demonstrated changes in the IC, as it was reported by human imaging studies that cocaine dependence is related to altered functional interactions of the IC with prefrontal networks.^56^

## 5 CONCLUSIONS

Compared with the extended cocaine IVSA, adolescent limited cocaine IVSA increased drug‐taking behaviors, indicating the importance of the developmental stage of initial drug exposure and the withdrawal period. The minor neuronal adaptations in the AAI may not be the primary neuronal substrate involved in high risk of relapse induced by adolescent brief exposure to cocaine. Future studies may refine the neuronal and molecular substrate explorations in the IC by investigating other IC subregions, other types of neurons such as GABAergic interneurons, changes in synaptic transmission, etc. Obviously, other brain regions, such as nucleus accumbens and mPFC, may be explored to identify the key neurological events which cause the limited drug exposure‐induced high drug‐taking behaviors.

## CONFLICT OF INTEREST

The authors report no conflicts of interest.

## Data Availability

The authors confirm that the data supporting the findings of this study are available (1) within this article and (2) from the corresponding author upon reasonable request.
